# Some Suggestions on Artificial Dentures

**Published:** 1909-01-15

**Authors:** W. A. Giffen

**Affiliations:** Detroit, Mich.


### SOME SUGGESTIONS ON ARTIFICIAL DENTURES.

BY W. A GIFFEN, D.D.S., DETROIT, MICH.

Read before the Michigan First District Dental Society, November 12, 1908.

Multiplicity of methods is the bane of our profession; in fact, I think too many methods are employed in taking impressions of the mouth, because, while this is one of the most important operations in the practice of dentistry, an accurate impression is the exception rather than the rule.

In describing the method I practice in taking an impression, I shall consider the taking of a partial impression for a partial denture, either upper or lower.

I select a full tray (Angle's design), large enough to pass over all the teeth remaining in the jaw and if the rim of the tray interferes with the soft tissues when the muscles of the lips and cheeks are contracted, I trim it off and make it smooth, so the plaster will not stick to it. I then rub a little vaseline, in which a small amount of wintergreen oil has been incorporated, over the inside of the tray and on the labial and buccal surfaces. I now mix a sufficient amount of plaster, place it in the tray, and carry it in position to the mouth. When the plaster commences to stiffen, I hold the tray firmly in position and ask the patient to try and displace it by contracting the muscles of the lips and cheeks. This forces the plaster close to the gums and has a tendency to compress any flabby areas of tissue which there may be. Any excess of plaster that has been compressed over the edge of the tray is now removed, and as soon as the plaster is hard I remove the tray, leaving the plaster in the mouth.

I remove the plaster by cutting grooves in suitable places, depending upon the location of the remaining teeth, and take it out in pieces, as few as possible, rinse them in water to wash off all small particles from the fractured surfaces, and allow them to dry. When the pieces are dry I put them together and secure with sticky wax on the outside of the impression. If the broken fragments need support, I lay one or two matches across the back of the impression and secure them to each fragment with wax.

There is no simpler or surer method of obtaining an accurate impression, as plaster (which undoubtedly is the best) is the only impression material used and the use of all forms of partial or special impression trays is dispensed with.

After the model is made, the mouth is again examined and all soft or spongy areas are marked on the model, and enough plaster scraped away so that the denture, when completed, will compress these areas, thus giving the denture firmer support in every way.

I use French's plaster, as it cuts rather easy, breaks with a sharp fracture, and sets rapidly.

In taking a full impression, I select a tray a little larger than the arch, and trim the edges so that it will not interfere with the soft tissues when the muscles of the lips and cheeks are contracted.

A sufficient amount of Perfection impression material, softened to the right consistency, is now placed in the tray and pressed to position in the mouth. The muscles of lips and cheeks are stretched, in turn, over the edge of the tray. This impression is now removed and all excess trimmed off with a sharp knife, as well as that portion filling any undercuts over the buccal or labial surfaces of the ridge.

I now have a tray that not only fits but compresses the tissues of all the flabby or spongy places, as I think they should be.

The inside of the tray is now coated with a little plaster, mixed thin, and again placed in position in the mouth. When the plaster begins to set I hold the tray firmly in position as with the partial impression, and have patient try to displace it by contracting the muscles of the lips and cheeks, thus forcing the plaster around edge of tray into any hollows over the ridge on the buccal or labial surfaces, and also compress any spongy areas of tissue there may be.

When the plaster is hard, remove the impression. The rim will break off when there are any undercuts, but can easily be replaced and waxed in position. By following this method I think we can get the most accurate impression possible.

### PLASTER CASTS.

When the impression has been prepared for pouring the cast, I make a matrix out of base plate wax, so that the vertical diameter of the model will be approximately the same as the horizontal diameter. I then attach the matrix to the impression.

We know that plaster expands a little during the hardening process (and like all other plastic materials which either contract or expand during this process) is liable to change shape, and more especially in the direction of the least resistance. So if the resistance in each direction is as nearly equal as possible, the danger of warpage is minimized.

There is no doubt in my mind that the reason so many so-called "rocky" plates have to be made over is because the cast was made too shallow and became warped during the setting process.

When I am ready to place the cast on the articulator, I simply saw off the portion I have no further use for.

I use Knickerbocker plaster, as it makes a very hard, strong cast if properly mixed.

Place plenty of water in the bowl and sift plaster in until it is about level with the surface of the water. Use a very narrow spatula and stir it just enough to mix it, because continued stirring causes too much air to become incorporated, with the result that the cast is not as dense or strong.

If there should be too much water, add more plaster; do not pour off any excess water, as some of the elements of crystallization which are in solution will be lost and the cast will not be so hard.

For vulcanite dentures I make temporary base plates of Ash's No. 7 soft metal. I cut a piece of metal to size and roughly shape it upon the plaster model, with the fingers, then swage it to place on the model with Ash's Flat Rubber Block Swager. It takes less time to make them, and they are far superior to trying in plates made from wax or any other plastic material. They do not change shape under the heat of the mouth, fit accurately, and are preferred by the patient. The models can also be kept in better condition, as they are always dry and there is no danger of getting them marred by hot wax.

These base plates form a proper foundation upon which to form rims of base plate wax to obtain the proper relation of the jaws to each other, for establishing lost facial contour, and all other steps necessary to obtain proper anatomic occlusion of artificial teeth.

Dr. J. H. Prothero, in concluding his treatment of this subject, in the March number of the Dental Review, says: "I am convinced that the system of anatomical occlusion of artificial teeth is now logical and practical and capable of every day application; that with each one now it is only a question of securing the proper appliances and working out the details in a practical manner. Further, that he who earnestly and sincerely makes an attempt along the lines mentioned, can not fail to see the utility as well as the simplicity of the method described, and will discard the plain line articulator, which is incapable of producing the most essential masticatory movements." Personally, I wish to add that the more I learn and understand his method, the more I think he is right.

Another good feature about the swaged soft metal base plate for upper dentures is that the natural contour of the soft tissues of the palatal surface is reproduced in the soft metal, which takes the place of wax until the invested case is separated, the result being a denture whose palatal surface is a perfect representation of the mouth, while the rubber is of uniform thickness.

In vulcanizing rubber dentures, tin foil should be burnished over the cast and also over the palatal surface of base plate, in order to get a proper finish.

Another nice way to finish a vulcanite denture is to line it with gold. To do this, take a piece of gold about 50 gauge, press it with the fingers to a rough fit on the model, remove and trim to size; replace gold lining, lay soft metal base plate over it and swage to place. Should the gold lining split under the pressure, lift off the base plate, place a piece of gold leaf over the split, and swage as before. Small retaining tags should be soldered to it, where necessary, so that it will be firmly attached to the rubber. This makes a very beautiful denture.

I am constructing a plate now, using platinum, gold and rubber, which I think will be very artistic, strong and clea n. I am using platinum, rolled very thin, as a lining, but instead of using retaining tags to attach it to the rubber, I have reinforced it with gold wire 18 gauge, around the edge, which of course makes a beautiful finish. I have also soldered an ornamental gold plate strengthener to the palatal surface, which also acts as a conductor of thermal change to the platinum lining. It will have all the advantages of a gold plate, be more beautiful, and rank second only to a continuous gum plate.

### GOLD DENTURES.

To make a double plate gold denture, secure a feasible metal die from the impression; cn this die swage a 30 gauge gold plate; solder finishing wire to edge. This plate is now used as a soft metal base plate until the teeth are articulated and all contouring of wax finished. Then slightly oil the lingual surface and pour a plaster core into it; when set, remove the core, make a matrix of plaster of Paris around it, and pour fusible metal over it. This gives a die of the lingual surface of the denture, into which the second or top plate is swaged.

The last plate swaged is now placed in position and the case flasked in order to prevent any alteration in the bite. The plates are then removed from the flask and soldered. The top plate should be left one-eighth of an inch shorter than the bottom plate, as this leaves a ledge at the distal end upon which pieces of solder are laid. Retaining tags are now soldered to the buccal and labial surfaces to give attachment to the rubber; it is again placed in the flask and the case is ready to pack, vulcanize and finish.

DISCUSSION.

DR. BUTTRICK: I was one of the committee that arranged the programme for the Society for the current year, and it was our endeavor to select subjects that would be of practical importance to each one of us. The subject for consideration this evening seems to me to be one of prime importance, although I am sure that it is not generally so considered,—in fact, when the subject of plate making is mentioned the average dentist begins to lose interest.

A Dental friend of mine told me that he once spoke in a rather slighting manner of the drudgery of plate-making to Dr. E. C. Moore, and he was somewhat startled at Dr. Moore's response—"Young man, the reason you do not like plate work is because you do not know anything about it." He said that he turned the matter over in his mind for a moment and was forced to the conclusion that Dr. Moore was right.

Dr. Giffen is one of those who has given a lot of time and thought to plate making and the Committee selected him, much against his wishes, as the man to handle the subject for us this evening.

To those of you who have listened to the paper, I am sure there has been much of practical importance. The subject is so large a one, however, that it cannot be covered in a single paper, and a dozen of them would not exhaust its possibilities. The portion of it that Dr. Giffen has touched upon has been thoroughly and comprehensively covered, and he has brought out many valuable points; when Dr. Giffen has finished, there is not much left to be said, so that I shall not go over this portion again, except, perhaps, to suggest that in my estimation a modeling compound impression for a lower denture is preferable to a plaster one, for the reason that a certain amount of compression of the soft tissues is desirable, and this one cannot secure with plaster only.

In using Knickerbocker plaster, I build a rim of wax about the impression; then, after wetting the impression thoroughly, I place a very small portion of the plaster, properly mixed, into the impression, and then jog it sharply until the plaster spreads thinly over the surface, thus bringing the bubbles to the top when they can be blown away; then a little more plaster is added and again jogged to place, and the bubbles blown away, and so on until the surface of the model has been built in densely, when it can be completed as rapidly as possible. This can easily be done, as the Knickerbocker model plaster sets very slowly.

Instead of the metal base plates, I use the Detroit Dental Manufacturing Co's "Perfection Base Plates," which can be readily softened in hot water, but become very hard upon cooling. Then, instead of taking a "Mash Bite" with these, I build the bite wax upon the ridges of the base plates and then trim them to proper size after which I seal them together in the mouth with a hot spatula and remove. This avoids the false relative position of the jaws so often gotten when the patient attempts to bite correctly through a mouth full of wax.

There is one thing, however, that I do not think the Doctor brought out with sufficient emphasis, and that is the subject of articulation. You can take perfect impressions, you can make perfect models, you can fashion perfect bite plates and take accurate bites, you can go on and turn out a beautiful and artistic looking denture, but if your articulation be faulty, the whole thing is not worth the price of the materials. In fact, I should consider the proper articulation of the teeth of chiefest importance, and it is right here that so many dentists fall down. The teeth must be arranged with proper relation to the ridge or fulcrum and with proper relation to each other, and should simulate, to an extent at least, the arrangement of the natural teeth in good occlusion. For this purpose one of the so-called anatomical articulators should be used, for the chopping motion of the old fashioned articulators is not enough. There are a number of very good ones upon the market, and with sufficient care and pains a very satisfactory and useful articulation can be secured. However, one can never hope to entirely equal nature, so that, under the most favorable conditions, the patient must learn to use the apparatus as supplied to him.

Beside that portion of plate making which may be considered as merely mechanical is the artistic side of it. In this the individuality of the operator has the chance to display itself. This relates to the proper choosing of the teeth that the size, shape and color may harmonize with the temperament and facial characteristics of the patient. In making a full denture, where none of the natural teeth remain as a guide, I select the shade, from my shade guide, which seems nearest to suit the case. Then I get a number of sets of teeth and roughly wax up the six anterior ones and place them in the patient's mouth, to see how they are going to look when in their proper position. If the first set does not look right, I try another, and so on until I am satisfied with the results. In seeing them in the mouth in this way, one can tell just what changes are necessary to get a more natural appearance than in seeing them out of the natural surroundings. Then an irregular arrangement of some of the anterior teeth will often produce a happy effect—such as lapping one central over the other, or lapping the laterals over the centrals, (a very common irregularity, by the way), or perhaps slightly spacing the teeth as one sometimes sees in life. Then, too, the judicious use of some of the Brewster mineral stains upon the anterior teeth will often give a natural effect.

Of course one cannot afford to spend a great deal of time upon plate work unless the patient is willing to pay the price necessary to compensate the dentist for the time consumed, but if the dentists, as a rule, were willing to give the time and thought to their plate-making that they do to some other branches of dentistry, they would be better paid for the plates they do make, and this much despised occupation would take a stand more in keeping with its real importance.

I would like to add: Dr Giffen says it is all right to patronize the laboratory. So I suppose it is; but I think if you take the time to finish the work yourself you get better results. A friend of mine puts in a lot of time to preparing his plates, he goes to all the trouble of getting the impressions and waxing them up, making his selections of the teeth, and then sends them over to the laboratory to be vulcanized and finished and many times when they come back they do not fit, something has been done to spoil the plates either in packing, vulcanizing or finishing. I always try them in the wax before I vulcanize them. When I have a difficult full set to make, I vulcanize one plate and try it in, and try the other in the wax before I vulcanize it. This gives me a chance to correct any fault in the occlusion.

DR. C. H. LAND: To discuss this subject in the light of the science of the matter it would be equivalent to writing a long paper; and if I should attempt to relate my experience or show some of the records of my own work in this line, it would be equivalent to covering more ground than the essayist has attempted.

I want to compliment our essayist for his courage in presenting this particular subject because our better dentists have neglected it; every student in college is going to be an operator—he is not going to make teeth if he can help it. Why? Because plate making has been so cheapened, that the art is lost. When I first went into dentistry we could do from $200 to $300 per month at $25 to $50 a set for rubber dentures. To-day I do not believe a first class practitioner would dare to charge even the minimum price. The styles of plates shown and referred to here have been made in many similar combinations of gold and platinum, gold and rubber, gold and aluminum, and other metals have been made for years. Metal dentures should be burnished over the plaster model to secure greater conformity to the mouth. If there is anything in the world that will train a man into successful prosthetic work it is making continuous gum work, because he cannot afford to make a mistake. Until I took up that subject and studied it scientifically throughout its various and complex details of construction, I did not make a complete success of continuous gum work. When I came to this city in 1871 I could not buy gold plate at the dental depots for making a set on gold; I had to go to the jeweler's to get it. There was but one man here then making continuous gum work, Dr. Geo. Field, and he was sending away to get it baked.

The idea of making a test trial plate on metal is an excellent one. I do not spend so much time taking articulations with articulators. The first thing I do in making an upper and lower denture is to take impressions of the mouth and get the casts and mount them in any kind of articulator by a mash bite, and approximate what I am going to do. Then I study the positions the upper teeth will take and their probable relations to the lower jaw, and I expect that in the large majority of cases the occlusion will not be correct. I make the upper denture with little regard to the lower one at first, and I complete it. I simply use the lower as an approximation of what I want finally. When I get the upper denture completed I have the prevailing expression of the face, width of the jaw, etc. I am not then afraid I will not then get lower one right. I then put the vulcanized and waxed lower in articulation again and complete the articulation. It is difficult to get patients to bite properly, if you tell a person to bite right they will not do it.

In adapting teeth, unless you first study the conditions of every mouth, how much depth there is to the arch, how rigid that mouth is, study the soft parts and mark them out your cast, their relative thickness, etc., you are likely to be dissapointed in results. The regulation form of suction chamber, so called, and also the more recent patented articles I have no use for at all. All adhesion is governed by the angles and ridges surfaces of the individual mouth. If the mouth is flat as a board and exactly two inches in diameter, these would be only one method of estimating the retention of such a denture. If you are making a full set of teeth of all porcelain and have a very high ridge and a rigid mouth, I don't care how perfect your impression is, if you don't allow for its shrinkage latterly, when you get it done it will dig right into the side of the alveolar ridge and will not be a fit at all. If it was a perfectly horizontal plane, and it should shrink a quarter of an inch it would not make any difference. In proportion as the mouth goes away from the horizontal plane you are running against difficulties and the bearing of your denture must be different. These must be relieved on the horizontal portions and the ridges where the contact comes must be the positive points of support, then the whole plate surface will form to a contact and support and you have a scientifically fitted denture. If I have a mouth with a flat vault, with pronounced perpendicular alveolar ridges I put a rib on the side of the perpendicular wall, inside and outside, and then when the plate is inserted it is like a grip that holds on. With these perpendicular walls your smooth dentures slip and slide off, but put in your clincher dentures on these high walls and they fit every time—otherwise they will not fit at all. I would divide all dentures into friction and adhesive denures. Friction is a denture with a clasp of some sort; adhesive dentures are held in by adhesive saliva and close tcontact and not by atmospheric pressure. We have no such thing as plate retention by the exclusion of the air, because the saliva establishes an equilibrium. The true idea is to relieve the horizontal planes, especially on palatine surfaces, and secure all possible mechanical clinch.

Dr. Giffen: Dr. Buttrick is perfectly right when he says you cannot scrape a model exactly to represent the soft areas we may find in the mouth, but you do not have to scrape a cast made for a full denture if the impression has been taken as I described, as the stiff impression material used in making the tray will compress the soft tissues sufficiently. However, it is different when you make a cast from a plaster impression for a partial denture. The plaster does not compress the tissues sufficiently; so, by making soft areas or locating them on the cast you can scrape them down to approximate the shape the cast would be had there been no flabby or soft areas, and it is a fact that if the plate does not press a little too hard in those soft places the tissues will soon accomodate themselves to the denture. With plaster you can get an accurate impression of all irregular surfaces, but not with wax or perfection impression material, as it must change shape in order to come out. After the plate is finished it can easily be trimmed to go in where there are V-shaped spaces between the teeth.

Dr. Buttrick's method of taking the bite is the next best way, and I believe he gets good results; however, I think the metal base plates are better, as they are more rigid and will stay in position in the mouth better, and you have better adaptation than you can possibly get by moulding plastic material to the cast.

Regarding the use of stained teeth, I think they are very nice for suitable cases. I have used the S. S. White natural mould stained teeth on the platinum lined plate I showed you this evening. They serve the purpose well in full dentures, and for partial special cases very artistic results can be obtained by selecting the teeth and staining or coloring them with oil colors used for that purpose.

Regarding the assertion of Dr. Buttrick that he did not like to have a nice piece of work spoiled in a laboratory by having it turned over to a boy to finish. I know it happens occasionally, and when it does occur the chances are we kick a good deal harder than if we did it ourselves. Personally I like to finish up my own laboratory work. However, I know there are many dentists who do not like to make plates, and who are kept busy operating or doing other lines of work, or possibly that know they cannot do the work as well as their laboratory man will do it for them. I have sent plate work to laboratories occasionally myself, when I was too busy to get the work out on time. There can be no objection to your having your plate work done in this way, provided you can find some one to do it in the right way.

The cases I have shown you this evening were made by a student who never saw a vulcanizer until a year ago last September, and the only experience he has had is what he got in the mechanical laboratory at college, under Dr. McPhee, and a suggestion from myself occasionally. I consider the arrangement of the teeth on those cases to be correct. The carving of the wax to restore lost contour to be very artistic—in fact, they are correct in every particular.

The point I want to make is this: If you do send your plate work out, you should yourself first learn to know how a plate should be made. Start with an accurate impression and bite; explain to whoever does your laboratory work how you want the teeth articulated and the gums contoured and any other details you believe will make the work more beautiful or more comfortable to the patient. If you are going to have your laboratory work done by some one else, be very careful to select the right man and have an understanding with him as to how you want your work done, not cheaply, but right. Don't send a slip shod impression, a mash bite, and ask your laboratory man to select teeth for a patient he has never seen, and expect him to do all the work, furnish all materials, including teeth, for $2.50, and expect to get good results.

Now regarding the statement by Dr. Land that the anatomical articular and the scientific method of articulating teeth is all foolishness, I wish to say that I have not been doing this work so long as Dr. Land, and that we are not all so adept at articulating teeth as he says he is, and while I know that ability and experience overcome many obstacles, I cannot agree with him when he says any old hinge is a good enough articulator, and that the position and inclination of the teeth should be decided by the shape of the alveolar ridge. I have never heard of any dentist who claimed to be able to do successful work on that basis, except Dr. Land.

There is such a great variety in the shape of the alveolar ridge, due to absorption and other causes, that it seems to me to be a very unscientific method. Dr. Land states that lining plates is an old system. I know it is old, but at the same time we sometimes forget some of these old things that we ought to remember, and it is simply to remind you that they are here mentioned.

I have tried making undercuts on cases where I expected to have difficulty in retaining the plates in position, but with indifferent success, as I usually have to trim them off the plate before I can train my patient to become accustomed to them.

I have tried to bring out the more practical points about the plate work of every day practice. I shall be very glad to discuss continuous gum or porcelain dentures at another time, as I think there is nothing that compares with this style of plate. I am going to ask Dr. Land for permission to go to his office and spend some time in examining the continuous gum work he displayed here this evening.

I think I would rather have had more of the members take part in the discussion and offer suggestions, as I know I have a great deal to learn about making plates, I thought

I was pretty smart when I started to write this paper, and that I could give out a few useful ideas that all were not familiar with, but upon reviewing the literature upon this work and reading papers from such men as Dr. Prothero, Dr. Wilson and others, it made me feel that I would have done better if I had prepared a paper on what I do not know about artificial dentures. It does us all good to have these things brought forcibly to our minds occasionally, so the more a paper is criticised the more benefit we receive.

---

